# Pacing and Changes in Body Composition in 48 h Ultra-Endurance Running—A Case Study

**DOI:** 10.3390/sports6040136

**Published:** 2018-11-01

**Authors:** Beat Knechtle, Thomas Rosemann, Pantelis T. Nikolaidis

**Affiliations:** 1 Medbase St. Gallen Am Vadianplatz, 9001 St. Gallen, Switzerland; beat.knechtle@hispeed.ch; 2 Institute of Primary Care, University of Zurich, 8006 Zurich, Switzerland; thomas.rosemann@usz.ch; 3 Exercise Physiology Laboratory, 18450 Nikaia, Greece

**Keywords:** aerobic exercise, endurance performance, master athlete, running, body fat, body water

## Abstract

Pacing has been investigated in elite and master runners competing in marathon and ultra-marathon races up to 100 km and 100 miles, but not in longer ultra-marathons. In this case study, a 54-year-old master ultra-marathoner—intending to achieve as many kilometers as possible in a 48 h run—was examined. The changes in running speed during the race and selected anthropometric characteristics using bioelectrical impedance analysis (i.e., body mass and body water), during and after the race, were analyzed. The runner achieved a total distance of 230 km and running speed decreased non-linearly during the race. Body mass decreased, while percent body water increased, non-linearly, across the race. There was no statistically significant relationship between the decrease in body mass and the increase in percent body water. Considering the popularity of ultra-endurance running races, the findings of the present study offered valuable insight in the pacing and changes of body mass and body water during a 48 h run, and this information can be used by ultra-endurance runners and practitioners working with them.

## 1. Introduction

Pacing during endurance or ultra-endurance performance seems to have a considerable impact on overall performance. Pacing describes how an athlete distributes his work and energy throughout an exercise task [1]. In running, pacing strategies have been investigated for different distances, such as half-marathon,[2] marathon [3,4,5,6,7,8,9], and ultra-marathon distances [10,11,12,13], and for both master runners [3,6,7,8,9,11,13] and elite athletes [2,4,5].

In ultra-marathon running (i.e., any running distance longer than the marathon distance and/or longer in duration than 6 h), little data are available for pacing in both elite and master athletes [14]. For ultra-marathon races, mainly elite athletes have been investigated, competing in 100 km [10,11,12,13] and 100 mile [10] ultra-marathons. For master athletes (i.e., runners of 35 years and older), two studies investigated the pacing trends in 100 km ultra-marathoners [11,13] and, in one case study, the pacing of a 94-year-old runner in a 6 h run [15]. To the best of our knowledge, no further studies have ever investigated the pacing strategy of a master ultra-marathoner competing in any ultra-marathon longer than 6 h and/or 100 miles.

While the case study of the 94-year-old runner in a 6 h run also considered aspects of changes in anthropometric characteristics [15], we have only limited data about changes in anthropometric characteristics (e.g., body mass, body water) in ultra-marathoners competing in race distances longer than 100 km. It is well known that an ultra-marathon leads to a decrease in body mass [16,17,18]; however, the impact of a 48 h run has not been examined yet.

So far, changes in anthropometric characteristics have only been investigated by comparing pre-race and post-race values [16,17], with little data about the recovery period available [15]. Furthermore, we have no knowledge about changes in anthropometric characteristics during an ultra-marathon, since measurements during a race were lacking in the literature. Changes of body mass during a 24 h run have been examined in a laboratory setting in one study [19], which provided a well-controlled experimental setting for research, but was not ecologically valid and did not monitor the variation of body mass during recovery. A recent study was conducted looking at physiological responses to a 100 mile ultra-marathon [20].

Considering the increasing popularity of 48 h ultra-marathon running, the knowledge about pacing and acute physiological responses of body composition to this race, and post-exercise, would be of practical and theoretical interest for ultra-marathon runners and exercise physiologists, respectively [21]. Therefore, the aim of the present case study was (i) to investigate the pacing in a master ultra-marathoner in a 48 h ultra-marathon by analyzing his lap times, and (ii) to analyze changes in body composition (i.e., body mass, body water) during the race in 6 h intervals, and after the race, in 24 h intervals.

## 2. Materials and Methods

### 2.1. The Runner

Our runner is a 53-year-old recreational master runner (80.1 kg, 177 cm, BMI 25.6 kg/m^2^) who trains regularly and competes annually in several 6 h and 12 h ultra-marathons. While he completed, in 2017, three 24 h runs, he intended to run a 48 h ultra-marathon in 2018. In the preparation for the 48 h run, he changed nothing during training compared to the preparation for the 24 h run, except that he increased the weekly running volume in the last three months before the start from about 110 km to a maximum of 150 km without changing running speed. The training sessions during preparation were monitored by a Garmin GPS Smartwatch vívoactive™, which assisted the runner in maintaining a mean running speed of 8–9 min/km, equivalent to 7.0–7.5 km/h (intended race speed). The participant provided written informed consent, and the study was approved by the local institutional ethics review committee.

### 2.2. The Race

The runner started on 26 January 2018, in Athens, at the “13th Festival Athens Ultramarathon”, with the 48 h run [22]. The event included a 24 h run, a 48 h run, a 6 day run, a 1000 km run, and a 1000 mile run. This event is part of the IAU (International Association of Ultrarunners) [23]. The race takes place in the town of Glyfada, a suburb south of Athens. The area is located by the sea, and is in the immediate vicinity of the old airport.

In this run, all participants have to run as many laps as possible, with one lap measuring exactly 1.0 km and being completely flat. The rounds are counted electronically with a chip system, whereby the runners must weave two thin chips into their shoelaces of both shoes before the start. Each time the target is traversed, the distance actually completed appears on a large monitor for the orientation of the runners. The runner did not use a GPS watch to see his running speed while competing, but ran after the feeling he had acquired during the training runs. The goal was to achieve a distance of 40–42 km every six hours. To save energy for the second 24 h, he went for the first 24 h, so that he would run around 150 km, thus, about 10 km slower than in the 24 h run, with the aim of reaching 300 km.

The start time was on Friday at 17:00. Temperatures were a maximum of 15 °C during the day, and dropped to a minimum of 3 °C during the night. During the whole race time, there was no precipitation, and the sky was cloudless. Day and night, a strong wind blew from the north. The runner took care of the buffet of the organizer with water, coke, isotonic drink, energy bars, nuts, orange wedges, as well as with his own food, which consisted mostly of sweets such as chocolate. Twice a day, at lunch and dinner, there was a light hot meal provided by the organizer. Unfortunately, the offer of the organizer was very limited, and hot meals were missing completely during the cold nights. Thus, the performance of the runner deteriorated in the second 24 h as he had to take more and more breaks towards the end of the run, and partly had to insert long stages of marching.

### 2.3. Procedures

One hour before the start of the race, every 6 h during the race, immediately after the race, and then every 24 h for three days, we measured body mass and percent body water using the Tanita BC-545 Bioelectrical Impedance Scale (Tanita Corporation of America Inc., Arlington Heights, IL, USA) [24]. Energy intake and energy output were not measured, since it is well known that during long to very long endurance performances, the energy expenditure is higher than the energy intake, and an energy deficit arises [25,26]. Furthermore, the estimation of the energy expenditure during performance with the easy-to-use heart rate method, as a simple practical version for the competition, is relatively inaccurate [25,26]. The fluid intake was recorded in each lap.

### 2.4. Statistical Analysis

All data were expressed as mean and standard deviation. The acceptable type I error was set at *p* < 0.05. All statistical analyses were carried out using GraphPad Prism v. 7.0 (GraphPad Software, San Diego, CA, USA) and IBM SPSS v.23.0 (SPSS, Chicago, IL, USA). A linear regression analysis examined the variation of speed during race, and the relationship of body water with body mass. Pearson correlation coefficient *r* was used to examine the relationship among the abovementioned variables. Non-linear (4th grade) regression analysis examined the variation of anthropometric characteristics during the race and three days post-race. Mean speed was calculated for each hour of the race, and a one-way analysis of variance examined the main effect of hour of race on speed. Bonferroni post hoc comparisons examined differences among hour of races for speed. The magnitude of these differences was evaluated using partial eta square (*η*^2^*_p_*).

## 3. Results

**Figure 1:**
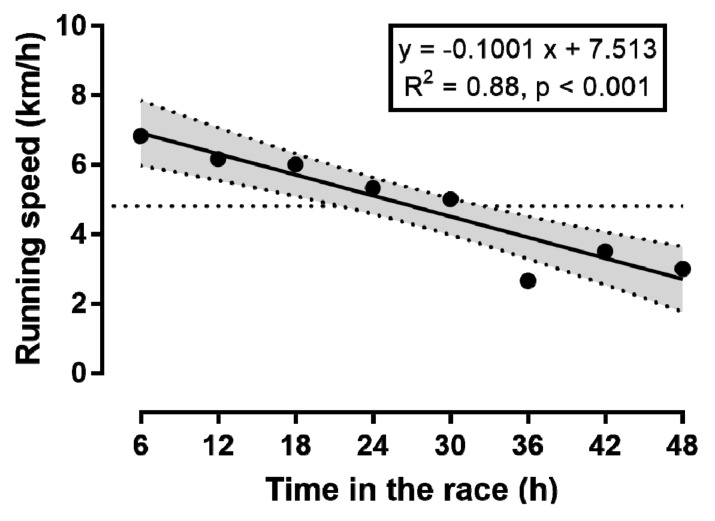
Change in running speed during the race, presented in 6 h intervals.

**Figure 2:**
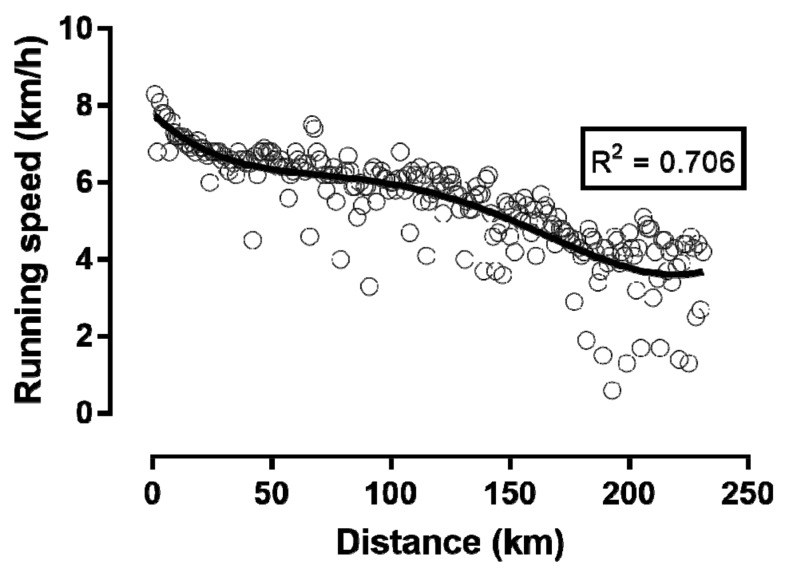
Change in running speed by lap during the completed distance.

**Figure 3:**
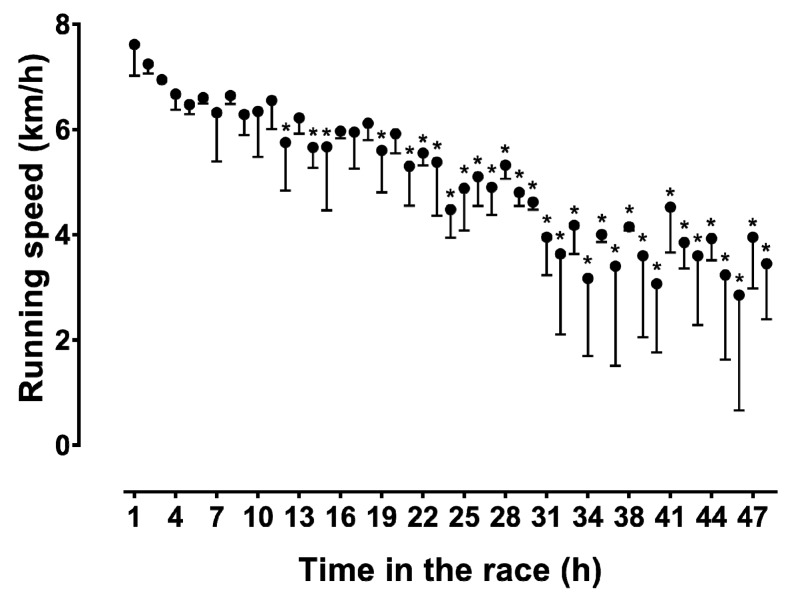
Change in running speed by lap during the completed time. * Different from running speed of the first hour. Error bars represent standard deviation.

The runner achieved—in contrast to his plan for 300 km—a total distance of only 230 km. When the average running speed is displayed in 6 h intervals, running speed decreased linearly (Figure 1). However, when we plot running speed per lap, as a function of the distance covered (Figure 2) and the elapsed time (Figure 3), then a non-linear decrease in running speed appears. A large main effect of hour of race on running speed was observed (*p* < 0.001, *η_p_*^2^ = 0.761), with the fastest speed in the 1st hour (7.61 ± 0.59 km/h) and the slowest in the 46th hour (2.85 ± 2.19 km/h). When we display running speed in this way, we see a sharp decrease in running speed within the first 6 h or during the first marathon. From the 6th to the 36th hour in the race (or until about the 170th km) the decrease in running speed was less pronounced. From the 37th to the 48th hour, running speed could even be maintained until the finish, with a small final end-spurt.

**Figure 4:**
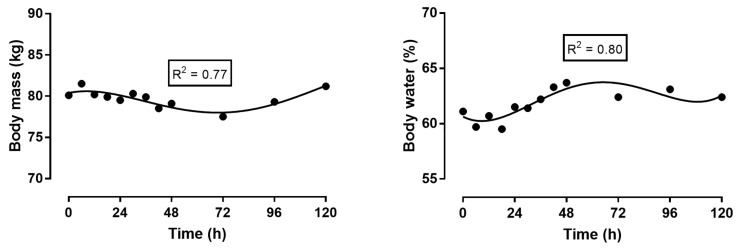
Change in body mass and percent body water during and after the race.

Regarding the variables measured using bioelectrical impedance analysis (BIA) (Figure 4), body mass decreased during the race, reaching its lowest value 24 h after the race, and then rising after the race. The percentage of body water increased, especially during the second half of the race.

**Figure 5:**
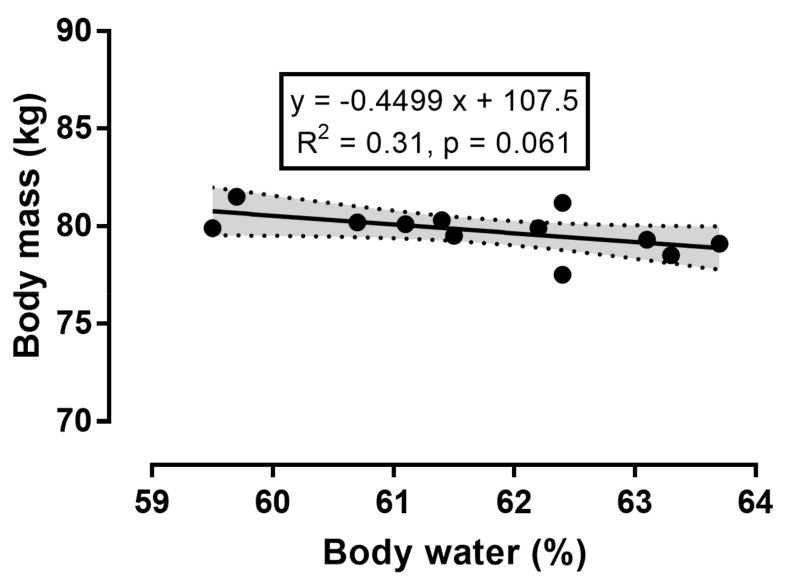
Correlation between percent body water and body mass.

Since the percentage of body water showed a counterbalancing course to body mass, we examined their correlation (Figure 5). There was no statistically significant association between body mass and percent body water (*r* = −0.56, *p* = 0.061). Nevertheless, it should be noted that their correlation approached statistical significance of a large magnitude.

**Figure 6:**
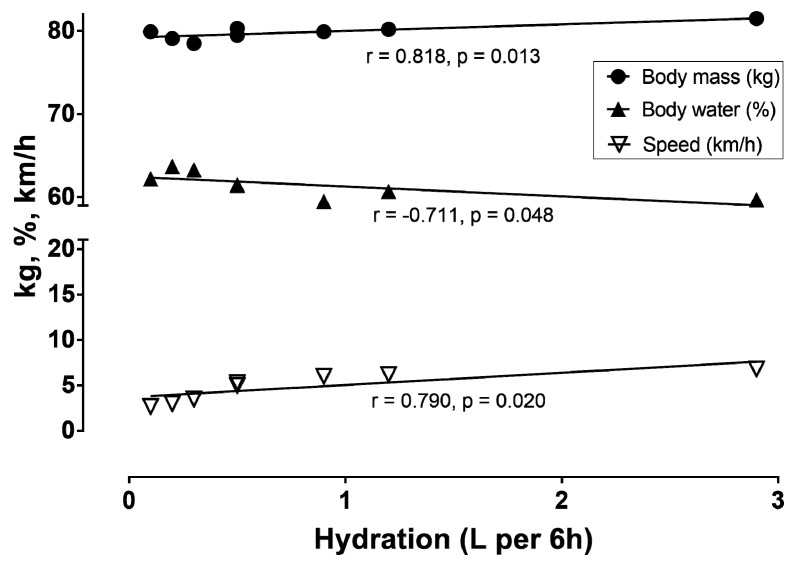
Correlation of hydration with body mass, body water, and speed.

Table 1 presents hydration, body mass, body water, and speed in 6 h intervals during the race. The correlations among these parameters can be seen in Figure 6.

**Figure 7:**
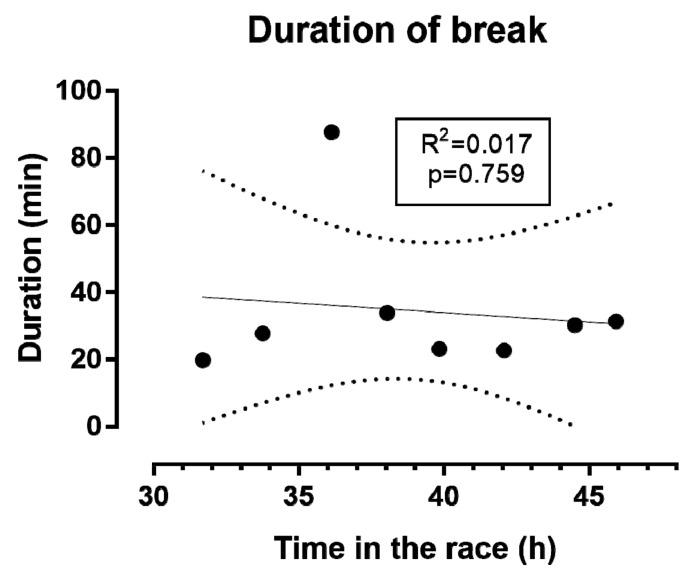
Occurrence of break during the race. Dashed lines represent 95% confidence intervals.

With regards to the breaks, the first one occurred at the 32nd hour of the race, after having completed 182 km (Figure 7). The breaks (*n* = 8) had a duration of 34:31 ± 22:01 (min:s), and ranged from 19:45 (min:s) to 1:27:41 (h:min:s).

## 4. Discussion

### 4.1. Decrease in Running Speed

We assumed that our runner could maintain the relatively low running speed, of just above the walking speed, constant over 48 h. When we now look at the running speed in 6 h intervals, running speed decreased linearly over time. However, when we look at the single laps, the running speed decreased non-linearly. A positive pacing strategy with a continuous decrease in speed during a race is commonly seen in ultra-endurance performances, such as in cyclists crossing the United States in the “Race Across America” [27], or in a cyclist in a self-paced world record attempt, in 24 h road cycling [28].

A possible explanation for the non-linear decrease in running speed could be the depletion of intramyocellular glycogen and lipids in the legs of the runner. The runner started with a relatively high running speed, and then the pace dropped relatively fast within a few hours. This first phase, with a fast drop in running speed, may have been due to the breakdown of intramuscular glycogen as a rapidly available source of energy [29]. In the further course of the race, the intra-myocellular lipids of the legs may have been used as another high-energy substrate [30], since these lipids are an important high-energy substrate for prolonged endurance exercise [31].

### 4.2. Change in Body Mass

Body mass decreased during the 48 h of the race. Often, the decrease in body mass is due to the decrease in body fat during an ultra-endurance performance [32,33], and the decrease in body mass during a race is related to race speed [32,33]. In the literature, there are several case reports and field studies of athletes who have competed in 24 h races [34,35,36,37]. However, there were only two case reports of cyclists where aspects of energy deficit [38] and a decrease in body fat [39] were examined in ultra-endurance performances lasting for 48 h. While in the one case, as expected, a pronounced energy deficit of more than 3000 kcal was detected [38], the other case showed a reduction in fat mass of 1 kg, with the greatest decrease being found between the 12th and 24th hours [39]. Since, in this second case, specific metabolites of lipid metabolism were examined using nuclear magnetic resonance at regular intervals in the urine, no steady state could be detected in the metabolism.

### 4.3. Changes in Body Water during the Race

BIA showed that body water initially decreased, then increased and remained elevated after the race. The increase in body water is most likely due to an expansion of plasma volume. This plasma volume expansion is observed during prolonged endurance exercise, such as a marathon [40], and is explained by a shift of protein into the intravascular compartment, as well as sodium retention by the kidney [41]. The measurements of body compartments using BIA are strongly dependent on body water. BIA induces a small current flowing throughout the body. This current measures the percentage of body water as a parameter for lean body mass [42]. It was surprising that that lean body mass in our runner increased after the race in the following days. Based on the results of the BIA, we assume that body water expanded into fat-free mass (lean body mass), and not into fat mass. During very long endurance exercise, body mass may increase [43], which can be attributed to an edema of skeletal muscle, as demonstrated, in one case, using DEXA (dual-energy X-ray absorptiometry) [44].

### 4.4. Limitations, Strength and Practical Applications

Considering the different acute physiological responses to ultra-endurance exercise varying for duration [45,46], the findings in the present study should be generalized with caution to other ultra-marathon races. Moreover, the specific physiological and training characteristics of the case study might be a limitation of this research. In addition, it has been shown that outcome measures of fat and fat-free mass, provided by BIA, might be misleading when the conditions of measurement varied across repeated measures. These conditions might include time of the day, body position, hydration, recent consumption of foods and drinks, the ambient and skin temperature, recent physical activity, and state of repletion of the urinary bladder. Recent studies analyzed the effect of physical exercise, and fluid loss through sweating and fluid intake [47] or food [48]. This was the first study conducted on pacing in a 48 h ultra-marathon and, as such, is a novel contribution to the literature.

## 5. Conclusions

In a master runner competing in a 48 h run, running speed decreased non-linearly during the race as a positive pacing. While body mass decreased, body water increased. Considering the popularity of ultra-endurance running races [49,50], the findings of the present study offered valuable insight in the pacing and changes of body mass and body water during a 48 h run and recovery, and this information can be used by ultra-endurance runners and practitioners working with them.

## Figures and Tables

**Table 1:** Hydration, and changes in body mass, body water, and performance, during the race.

Variables	Race Time (h)							
	6	12	18	24	30	36	42	48
Hydration (L)	2.9	1.2	0.9	0.5	0.5	0.1	0.3	0.2
Body mass (kg)	81.5	80.2	79.9	79.5	80.3	79.9	78.5	79.1
Body water (%)	59.7	60.7	59.5	61.5	61.4	62.2	63.3	63.7
Speed (km/h)	6.8	6.2	6.0	5.3	5.0	2.7	3.5	3.0
